# https://botryosphaeriales.org/, an online platform for up-to-date classification and account of taxa of Botryosphaeriales

**DOI:** 10.1093/database/baab061

**Published:** 2021-10-15

**Authors:** N a Wu, Asha J Dissanayake, Ishara S Manawasinghe, Achala R Rathnayaka, Jian-Kui Liu, Alan j.l Phillips, Itthayakorn Promputtha, Kevin D Hyde

**Affiliations:** CAS, Key Laboratory for Plant Diversity and Biogeography of East Asia, Kunming Institute of Botany, Chinese Academy of Sciences, Kunming 650201, P.R. China; Center of Excellence in Fungal Research, Mae Fah Luang University, Chiang Rai 57100, Thailand; School of Life Science and Technology, Center for Informational Biology, University of Electronic Science and Technology of China, Chengdu 611731, P.R. China; School of Life Science and Technology, Center for Informational Biology, University of Electronic Science and Technology of China, Chengdu 611731, P.R. China; Innovative Institute for Plant Health, Zhongkai University of Agriculture and Engineering, Guangzhou 510225, P.R. China; Center of Excellence in Fungal Research, Mae Fah Luang University, Chiang Rai 57100, Thailand; School of Life Science and Technology, Center for Informational Biology, University of Electronic Science and Technology of China, Chengdu 611731, P.R. China; Biosystems and Integrative Sciences Institute (BioISI), Universidade de Lisboa, Lisbon, 1749-016, Portugal; Department of Biology, Chiang Mai University, Chiang Mai 50200, Thailand; CAS, Key Laboratory for Plant Diversity and Biogeography of East Asia, Kunming Institute of Botany, Chinese Academy of Sciences, Kunming 650201, P.R. China; Center of Excellence in Fungal Research, Mae Fah Luang University, Chiang Rai 57100, Thailand; Innovative Institute for Plant Health, Zhongkai University of Agriculture and Engineering, Guangzhou 510225, P.R. China; Department of Biology, Chiang Mai University, Chiang Mai 50200, Thailand

## Abstract

Fungi are eukaryotes that inhabit various ecosystems worldwide and have a decomposing effect that other organisms cannot replace. Fungi are divided into two main groups depending on how their sexual spores are formed, viz. Ascomycota and Basidiomycota. The members of Botryosphaeriales (Dothideomycetes, Ascomycota) are ubiquitous. They are pathogenic on a wide range of hosts, causing diverse diseases including dieback, canker, leaf spots and root rots and are also reported as saprobes and endophytes worldwide. As an important fungal group, of which most are plant pathogens, it is necessary to organize data and information on Botryosphaeriales so that scientific literature can be used effectively. For this purpose, a new website, https://botryosphaeriales.org is established to gather all published data together with updates on the present taxonomy of Botryosphaeriales. The website consists of an easy-to-operate searching system and provides an up-to-date classification together with accounts of Botryosphaeriales taxa, including colour illustrations, descriptions, notes and numbers of species in each genus, as well as their classification. Thus, readers will be able to obtain information on botryosphaerialean taxa through this platform.

Database URL: https://botryosphaeriales.org/

## Introduction

Botryosphaeriales (Dothideomycetes) was established in 2006, to accommodate a single family, Botryosphaeriaceae (1). Nine families—Aplosporellaceae, Botryosphaeriaceae, Endomelanconiopsisaceae, Melanopsaceae, Phyllostictaceae, Planistromellaceae, Pseudofusicoccumaceae, Saccharataceae and Septorioideaceae—were accepted in Botryosphaeriales by Wijayawardene *et al.* (2). However, based on morpho-molecular analyses and evolutionary divergence times, the number of families accepted in Botryosphaeriales was reduced to six, namely Aplosporellaceae, Botryosphaeriaceae, Melanopsaceae, Phyllostictaceae, Planistromellaceae and Saccharataceae (3, 4). Endomelanconiopsisaceae and Pseudofusicoccumaceae were synonymized under Botryosphaeriaceae and Phyllostictaceae, respectively, while Septorioideaceae was synonymized under Saccharataceae (3). Members of Botryosphaeriales are found worldwide, on many different host plants (5–17). They are endophytes, pathogens and saprobes and as opportunistic pathogens, they are of considerable importance to agriculture, horticulture and forestry (18, 19). They cause severe diseases of economically important crops and plants leading to huge economic losses (20, 21). *Botryosphaeria, Diplodia, Dothiorella, Lasiodiplodia, Neofusicoccum* and *Phyllosticta* are the major pathogenic genera in Botryosphaeriales. *Botryosphaeria dothidea, Diplodia seriata, Lasiodiplodia theobromae* and *Neofusicoccum parvum* are associated with grapevine dieback worldwide (27). *Diplodia seriata* has been reported as a pathogen on a wide range of hosts including eucalyptus, pine and stone fruits (22–26). *Phyllosticta citricarpa* and *P. citriasiana* cause freckle on banana and brown spots on pomelo, respectively (6, 9). One species can occur on several hosts in the same country, and pathogenicity of a species can vary from one region to another (19). This reflects the importance of gathering data on these fungal taxa to understand disease epidemiology.

Aplosporellaceae, Melanopsaceae and Planistromellaceae have immersed or semi-immersed multiloculate ascostromata, while those of Botryosphaeriaceae, Phyllostictaceae and Saccharataceae are uniloculate (3, 8, 28, 29). Asci are bitunicate, with a thick endotunica (3, 8, 28, 29). Ascospores are hyaline or pigmented, septate or aseptate, ellipsoid to ovoid. Conidiomata are pycnidial, uni- to multilocular, frequently embedded in stromatic tissue. Conidiogenous cells are hyaline. Conidia are hyaline or pigmented, septate or aseptate, thin- or thick-walled (3, 8, 28–32).

All newly published data, which usually provide detailed descriptions and illustrations of new records, new species, new genera or new families, will be used to update the database. None of the papers generally link data from all members of the order Botryosphaeriales. Up until now, some websites involving specific groups of fungi have been established, such as http://www.facesoffungi.org (33), https://onestopshopfungi.org (34), http://www.marinespecies.org (35), https://fungalgenera.org (36), https://www.dothideomycetes.org (37) and https://sordariomycetes.org (38). However, there is no database on Botryosphaeriales. Therefore, in this paper, we provide a search method, designed to gather all the information about Botryosphaeriales into one website.

## The need for a Botryosphaeriales database

Many Botryosphaeriales species are associated with diseases in branches, leaves, fruits and seeds of aquatic and terrestrial plants (20, 31, 32, 39–43). In recent years, in-depth studies on Botryosphaeriales have resulted in changes in the genus/family-level classification (2–4). Moreover, the number of publications and research works related to Botryosphaeriales species are increasing all over the world (18, 20, 21, 44–48). With the increased number of studies on morphology, ecology and especially DNA-based phylogenetics, more and more new species are constantly being discovered. However, there are still many aspects needing clarification, such as naming new species from environmental samples, resolving the opportunistic pathogenic nature and how to define species boundaries in Botryosphaeriales.

Currently over 2300 species have been described in Botryosphaeriales in MycoBank based on morpho-molecular evidence (4). Most of the members of this order have been scientifically documented. These publications on *Botryosphaeriales* comprise various aspects such as taxonomy, morphology, pathogenicity or evolutionary studies. Information on Botryosphaeriales taxa are scattered in over 1000 publications mainly as books and research papers. Hence, the intention of this website is to gather all data regarding taxa accepted in Botryosphaeriales into a single entity that can be updated as new information becomes available.

The present website, https://botryosphaeriales.org/, focuses on the Botryosphaeriales with the following objectives: (i) gather all scattered data of accepted Botryosphaeriales taxa into a single platform, (ii) provide notes on the recent changes in genera and species of Botryosphaeriales with updated taxonomy and phylogeny and (iii) provide a list of all literature related to Botryosphaeriales. Hence, this website will be the best platform to access information on the botryosphaerialean taxa easily with simple searches, thus reducing the time spent on searching for information.

## The Botryosphaeriales website

Botryosphaeriales.org is a website dedicated to Botryosphaeriales taxa by providing an up-to-date account with descriptions, colour illustrations, culture characteristics, associated hosts, distribution and notes for order, families, genera and species. The website, https://botryosphaeriales.org will be updated periodically, keeping abreast of current literature. It is convenient for all mycologists and pathologists who need information about the history and current classification status of botryosphaerialean taxa. The home page of the website has a mailbox so that mycologists, pathologists or anyone else who use this website can suggest ideas to improve it.

## Construction

All fungi in the Botryosphaeriales.org are listed according to the latest classification (3, 4, 49). Each entry includes the accepted binominal name, Index Fungorum number, Faces of Fungi number, MycoBank number, ex-type culture collection number, dry culture collection and herbarium number and GenBank accession numbers of available DNA sequences. This website is handled and inspected regularly by the head curator, managing curator, senior curator and three other curators with expertise on botryosphaerialean taxonomy and phylogeny (Table 1).

**Table 1.** List of curators for *Botryosphaeriales* webpage

Position	Name	Affiliation	Contact details
Head Curator	Alan J.L. Phillips	Microbiology and Biotechnology Laboratory Biosystems and Integrative Sciences Institute Faculty of Science, University of Lisbon Campo Grande, 1749-016 Lisbon, Portugal	alan.jl.phillips@gmail.com
Managing Curator	Na Wu	Center of Excellence in Fungal Research, School of Science, Mae Fah Luang University, 333 Moo 1 Muang District, Chiang Rai 57100, Thailand	wuna220@gmail.com
Senior Curator	Asha J. Dissanayake	School of Life Science and Technology, University of Electronic Science and Technology of China, Chengdu 611731, P.R. China	asha.janadaree@yahoo.com
Curators	Kevin D. Hyde	Center of Excellence in Fungal Research, School of Science, Mae Fah Luang University, 333 Moo 1 Muang District, Chiang Rai 57100, Thailand	kdhyde3@gmail.com
	Ishara S. Manawasinghe	Innovative Institute for Plant Health, Zhongkai University of Agriculture and Engineering, Guangzhou 510225, P.R. China	ishara.ishara@yahoo.com
	Achala R. Rathnayaka	Center of Excellence in Fungal Research, School of Science, Mae Fah Luang University, 333 Moo 1 Muang District, Chiang Rai 57100, Thailand	rathnayakaachala@gmail.com

## Database interface and visualization

The website can be accessed at URL https://botryosphaeriales.org and comprises eight main headings, i.e. Home, Taxa, Archives, Curators, History, References, Notes and Contact. The interface is user-friendly, and each section comprises the following information.

### Home

**Figure 1.**
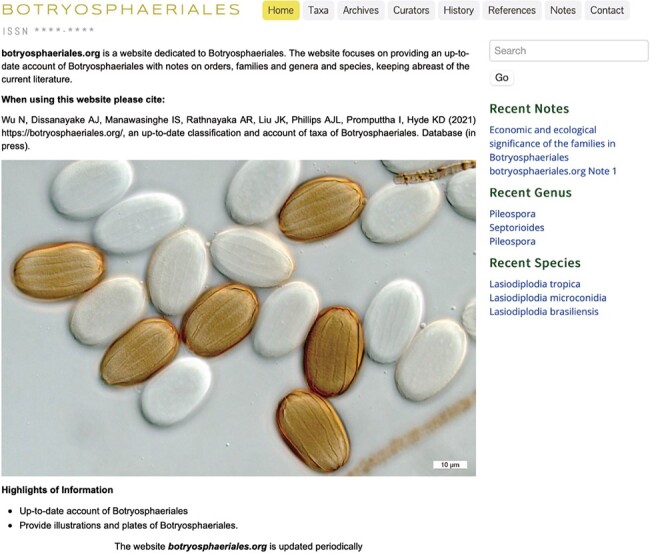
The homepage of Botryosphaeriales webpage.

**Figure 2.**
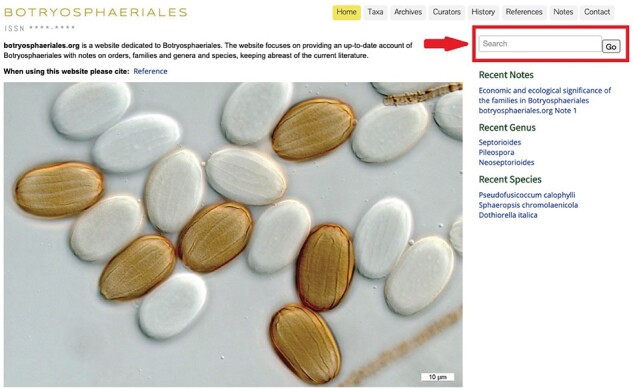
Red arrow indicates search tool to enter the taxon name.

**Figure 3.**
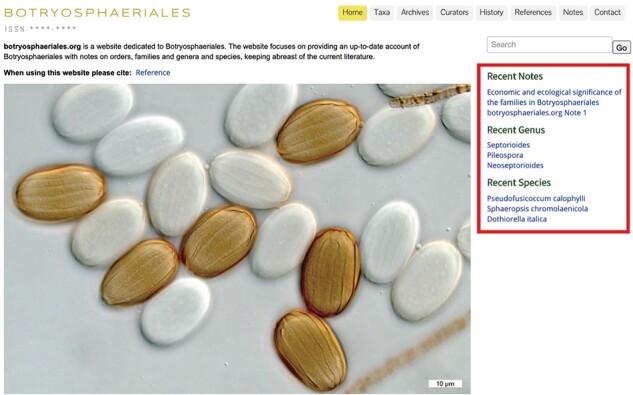
The recent notes, genus and species to enable easy access.

The homepage briefly introduces the objectives of the website and has links to highlights of information. Colour photos of Botryosphaeriales are displayed representing the key morphological features of this order (Figure 1). The relevant information can be accessed easily using the search box located at the right top corner (Figure 2). Beneath the search box, there is quick access to recent notes, recent genera and recent species. Clicking on these options opens the link in a new window (Figure 3).

### Taxa

**Figure 4.**
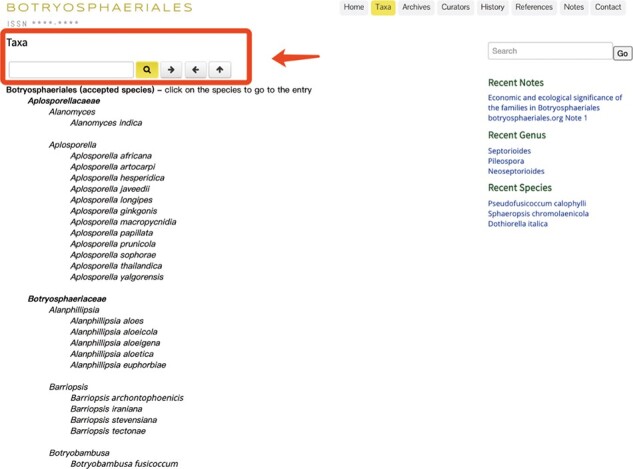
The ‘Taxa’ view of the Botryosphaeriales webpage.

**Figure 5.**
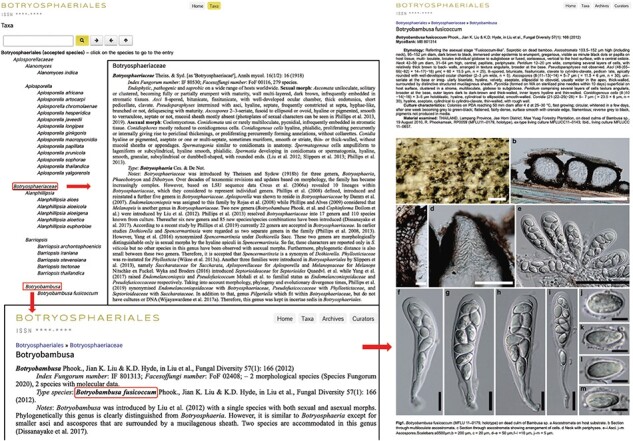
Details of the genus *Botryobambusa.*

Under the ‘Taxa’ heading, the recent taxonomic classification as families, genera and species of Botryosphaeriales is provided (Figure 4). In addition, an updated phylogenetic tree for the order is given at the bottom. The search box at the top left corner will facilitate quick access to each entry on the website. Clicking a listed species name will direct to the full description and details in the archives section (Figure 5).

### Archives

**Figure 6.**
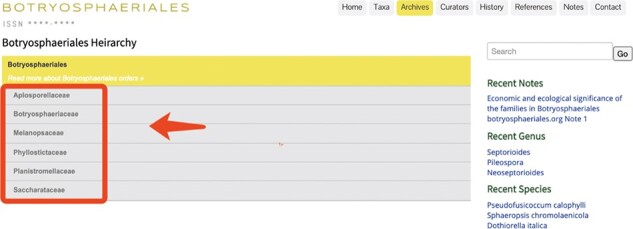
Appearance of the ‘Archives’ section in Botryosphaeriales webpage.

**Figure 7.**
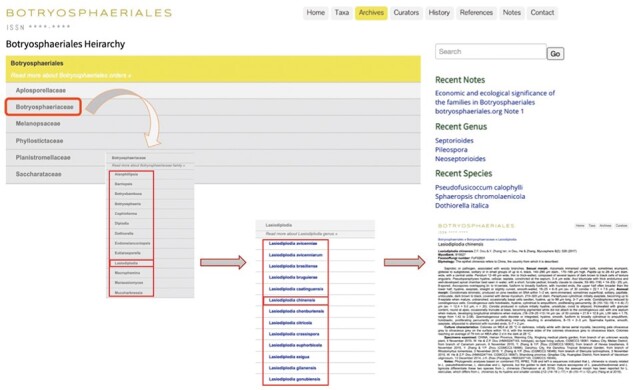
User-friendly interface of the ‘Archives’ section in Botryosphaeriales webpage.

**Figure 8.**
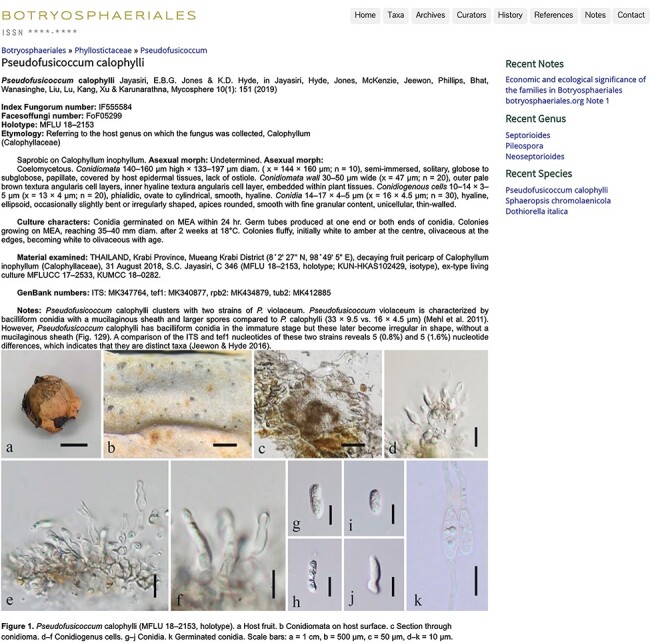
An entry with description, notes and plate (15).

The ‘Archives’ is the main section containing all information on every entry in the website (Figure 6). It consists of six tabs representing each family in Botryosphaeriales. Clicking on a particular family will automatically generate a dropdown list including all the genera belonging to the particular family (Figure 7). This can be further expanded into genus and species levels (Figure 7). By clicking the link for each species will open a new window containing all information on that species (Figure 8).

### Curators

**Figure 9.**
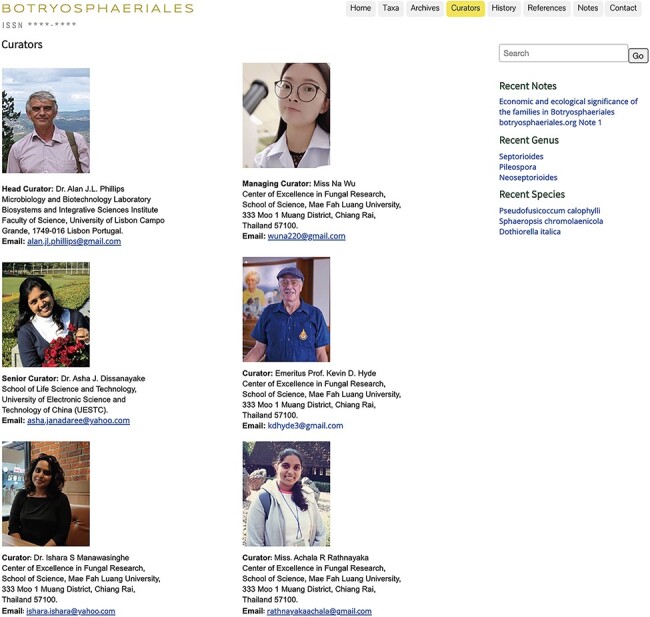
Curators of the Botryosphaeriales webpage.

This section provides information about the curators who handle and update the website (Table 1; Figure 9).

### History

This section provides a brief account of the classification of Botryosphaeriales.

### References

A list of the citations used in the entries and history as well as other related information are provided under this heading.

### Notes

Economic and ecological significance of families in Botryosphaeriales are given in this section. In addition, trends and current applications of the species of Botryosphaeriales will be added regularly to this section.

### Contact

This section provides the contact details of the website and allows users to communicate any comments and suggestions.
